# Increased generation of Foxp3^+^ regulatory T cells by manipulating antigen presentation in the thymus

**DOI:** 10.1038/ncomms10562

**Published:** 2016-02-29

**Authors:** Jiqiang Lin, Lu Yang, Hernandez Moura Silva, Alissa Trzeciak, Yongwon Choi, Susan R. Schwab, Michael L. Dustin, Juan J. Lafaille

**Affiliations:** 1 Molecular Pathogenesis Program, Kimmel Center for Biology and Medicine at the Skirball Institute, New York University School of Medicine, 540 First Avenue, New York, New York 10016, USA; 2 The Sackler Institute of Graduate Biomedical Sciences, New York University School of Medicine, New York, New York 10016, USA; 3 Department of Pathology and Laboratory Medicine, Institute for Immunology, University of Pennsylvania Perelman School of Medicine, Philadelphia, Pennsylvania 19104, USA; 4 Department of Pathology, New York University School of Medicine, New York, New York 10016, USA; 5 Nuffield Department of Orthopaedics, Rheumatology and Musculoskeletal Sciences, Kennedy Institute of Rheumatology, University of Oxford, Oxford OX3 7FY, UK

## Abstract

Regulatory T-cell (Treg) selection in the thymus is essential to prevent autoimmune diseases. Although important rules for Treg selection have been established, there is controversy regarding the degree of self-reactivity displayed by T-cell receptors expressed by Treg cells. In this study we have developed a model of autoimmune skin inflammation, to determine key parameters in the generation of skin-reactive Treg cells in the thymus (tTreg). tTreg development is predominantly AIRE dependent, with an AIRE-independent component. Without the knowledge of antigen recognized by skin-reactive Treg cells, we are able to enhance skin-specific tTreg cell generation using three approaches. First, we increase medullary thymic epithelial cells by using mice lacking osteoprotegerin or by adding TRANCE (RANKL, Tnfsf11). Second, we inject intrathymically peripheral dendritic cells from skin-draining sites. Finally, we inject skin tissue lysates intrathymically. These findings have implications for enhancing the generation of organ-specific Treg cells in autoimmune diseases.

## Abstract (web summary)

The degree of Treg self-antigen reactivity varies across experimental systems. Here the authors develop a new model of skin autoimmunity and show that the size of tissue-specific Treg pool in the thymus depends on AIRE, the availability of the tissue antigen, and its presentation by dendritic cells.

## Graphical abstract

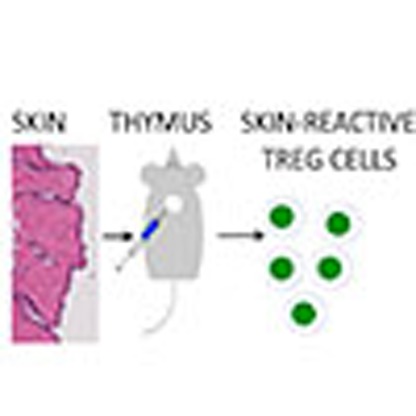

Regulatory T cells (Tregs) expressing FOPX3 are indispensable for the maintenance of immune homeostasis and self-tolerance^1^,^2^,^3^. T-cell receptor (TCR) specificity is generally considered to play an instructive role in thymus-generated Treg (tTreg) cell differentiation, as supported most directly by TCR transgenic RAG-deficient mice^4^,^5^. TCR transgenic mice made from conventional T cells (Tconvs) do not generate tTreg unless their cognate antigens are ectopically expressed in the thymus^6^,^7^,^8^. In contrast, TCR transgenic mice made using TCR from Treg cells display natural generation of Treg cells, although in small numbers, without any antigen manipulation^9^,^10^,^11^. It was concluded that although TCR is instructive for Treg cell selection, the size of a tTreg clone in the thymus was limited by a small tTreg-inducing niche^9^,^11^.

The ultimate goal of this study is to improve the generation of antigen-specific Treg cells by manipulating antigen presentation in the thymus. Towards that end, we used TCR transgenic mice expressing a Treg TCR that recognizes an antigen present in the skin. Elimination of the Foxp3 programme in these mice resulted in autoimmune skin inflammation. The antigen recognized by this Treg TCR is expressed by medullary thymic epithelial cells (mTEC), with a high percentage of Treg cells developing in an autoimmune regulator (AIRE)-dependent manner but also a small population of Tregs that develop in an AIRE-independent manner. Both AIRE-dependent and AIRE-independent Treg development required re-presentation by bone marrow (BM)-derived antigen-presenting cells (APCs).

Our studies suggest that clonal Treg selection is limited by the amount of high-affinity thymic (agonist) ligands and indicate a way to increase organ-specific Treg populations.

## Results

### 2P24 Treg TCR recognizes an antigen expressed in the skin

To study the key factors that limit Treg cell selection, we used TCR transgenic mice expressing TCR obtained from FOXP3^+^ Treg cells of unknown specificity^9^. We focused on two TCR clones, A12 and 2P24. A12 mice were derived from a thymic Foxp3^+^ Treg cell and 2P24 mice were derived from a Foxp3^+^ Treg cell found in pooled peripheral lymph nodes^9^. Unless otherwise stated, all TCR transgenic mice have been crossed to Rag1^−/−^ mice. Given the extremely high frequency of progenitors with the same TCR in TCR transgenic mice, we ensured that our conclusions about Treg development were confirmed in conditions of low precursor frequency, normal thymic anatomy and adequate timing of the expression of the TCRα chain^9^.

To test the hypothesis that the niche for Treg selection in 2P24 TCR transgenic mice (hereafter referred to as 2P24 mice) and A12 TCR transgenic mice (hereafter referred to as A12 mice) was limited by the amount of specific ligands available in the thymus, we studied the origin of the antigens recognized by our Treg TCR.

**Figure 1:**
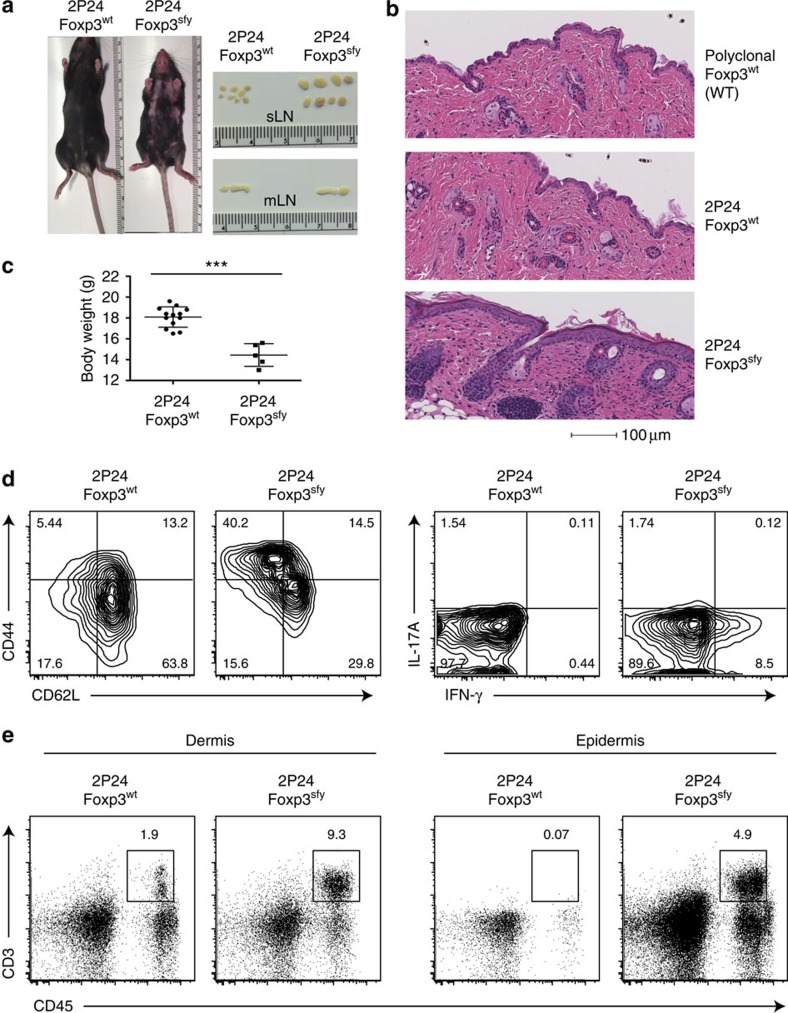
2P24 Treg TCR recognizes an antigen expressed in the skin. (**a**) Focal alopecia and enlarged skin-draining lymph node (LN) in 2-month-old 2P24 Foxp3^sfy^ mice. (**b**) Haematoxylin and eosin (H&E) staining of skin from 2-month-old WT mice, 2P24 Foxp3^wt^ and 2P24 Foxp3^sfy^ mice. (**c**) Body weight of 2-month-old 2P24 Foxp3^wt^ and 2P24 Foxp3^sfy^ littermate mice. (**d**) CD44, CD62L, interferon (IFN)-γ and IL-17A expression in CD4^+^ T cells from skin-draining LN of 2P24 Foxp3^wt^ and 2P24 Foxp3^sfy^ mice. (**e**) Lymphocyte infiltration in dermis and epidermis of 2P24 Foxp3^wt^ and 2P24 Foxp3^sfy^ mice. Data are representative of two independent experiments with three to five mice per group. Statistics were performed with unpaired Student's *t*-test. Error bars represent s.e.m. *P*-values >0.05: nonsignificant (NS); ***P-values ≤0.001: significant.

As reported earlier, all our Treg TCR transgenic mice, both RAG deficient and sufficient, remained healthy under steady state, showing no signs of autoimmune diseases. As our Treg TCR mice harboured considerable number of Treg cells in the peripheral lymphoid organs^9^, it was possible that those Treg controlled the activation of the remaining FOXP3^−^ T cells of identical specificity, preventing the development of autoimmune diseases. To assess the pathogenicity of the Treg TCR in the absence of Tregs, we crossed 2P24 and A12 TCR Tg with Foxp3-deficient mice (Foxp3^sfy^). Strikingly, all 2P24 Foxp3^sfy^ mice readily manifested signs of autoimmune disease in the skin, with severe inflammation, crusting of eyelids, footpad and tail, and accompanied by a degree of hair loss (Fig. 1a and Supplementary Fig. 1a). Histology analysis showed extensive cell infiltration at the skin and thickening of the epidermis, whereas no abnormalities were observed in the lungs, liver and intestines (Fig. 1b and Supplementary Fig. 1b). We also observed lymphadenopathy, which, strikingly, was limited to the skin-draining lymph nodes (Fig. 1a). Compared with Foxp3^wt^ littermates, 2P24 Foxp3^sfy^ mice were significantly smaller and had decreased body weight (Fig. 1c). Splenic T cells from 2P24 Foxp3^sfy^ mice lost their naive phenotype and showed increased interferon-γ production compared with 2P24 Foxp3^wt^ (Fig. 1d). There was increased T-cell infiltration in the skin, with a more dramatic change in the epidermis (Fig. 1e). The cross with Foxp3^sfy^ is not pathogenic *per se*, as we have previously crossed ovalbumin (OVA)-specific TCR transgenic RAG1^−/−^ mice with Foxp3^sfy^ mice and showed no spontaneous disease^12^. The spontaneous disease in 2P24 Foxp3^sfy^ mice supports the autoreactive nature of the 2P24 Treg TCR.

The 2P24 TCR is strictly restricted by H-2^u^ maor histocompatibility complex (MHC); in H-2^b^ mice, there is a complete arrest at the CD4/CD8 double positive (DP) stage (Supplementary Fig. 2). We took advantage of the high sensitivity of H-2^b^ 2P24 DP-arrested cells to H-2^u^/peptide stimulation, to study the skin reactivity of the 2P24 TCR. One of the earliest signs of TCR activation is the upregulation of CD69, which remains at low levels in DP cells from 2P24 H-2^b^ mice (Supplementary Fig. 2). However, when these DP cells were exposed to H-2^u^ APCs, there was a significant upregulation of CD69 expression, which was most prominent in the presence of skin-draining dendritic cells or splenic cells supplemented with skin lysates (Supplementary Fig. 3). Thus, the 2P24 TCR, initially obtained from a randomly selected Foxp3^+^ Treg cell, efficiently responds to antigens present in the skin.

We also crossed the A12 Treg transgenic TCR mice with Foxp3^sfy^. However, in this case, we did not see any overt autoimmune pathology (Supplementary Fig. 1c). It remains possible that a pathogenic self-reactivity exists but is not apparent in the tissues that we examined.

### AIRE-dependent and -independent development of Treg cells

The fact that the skin-reactive 2P24 clone gave rise to tTreg cells suggested that these developing 2P24 cells could have encountered, in the thymus, high-affinity ligands, which are related to the skin. AIRE is known to enhance the expression of tissue-restricted antigens (TRAs) in thymic medullary epithelial cells^13^,^14^,^15^.

**Figure 2:**
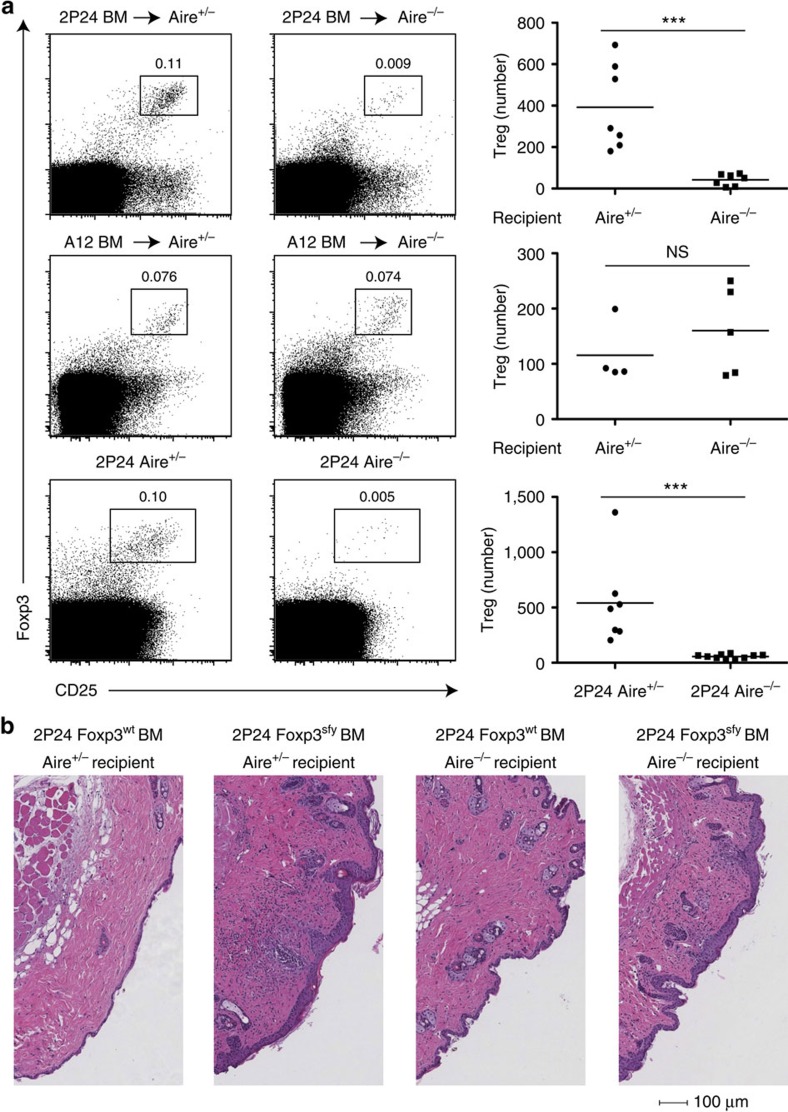
AIRE-dependent and -independent development of Treg cells. (**a**) Top row, three panels: CD45.2 2P24 BM cells were transferred into lethally irradiated CD45.1 Aire^+/−^ or Aire^−/−^ recipients. Eight weeks later, thymi were analysed by flow cytometry. Left two panels: representative staining of CD45.2^+^CD4SP cells; right panel: Treg numbers per total thymi. Results are summary of three independent experiments. Middle row three panels: same as top row three panels but using BM cells from CD45.2 A12 mice. Results are summary of three independent experiments. Bottom row, three panels: 2P24 crossed to Aire^−/−^ mice. Left two panels: representative staining of CD4SP cells from thymi of 2-month-old mice; right panel: Treg number per total thymi. Results are summary of three independent experiments. (**b**) 2P24 Foxp3^wt^ BM cells or 2P24 Foxp3^sfy^ BM cells were transferred into sublethally irradiated Rag1^−/−^ Aire^+/−^ or Rag1^−/−^ Aire^−/−^ recipients. Shown are haematoxylin and eosin (H&E) staining of the skin from recipients 10 weeks after transfer. Data are representative of two independent experiments with three to four mice per group. Statistics were performed with unpaired Student's *t*-test. Error bars represent s.e.m. *P*-values >0.05: nonsignificant (NS); *P-values ≤0.05: significant; **P-values ≤0.01: significant; ***P-values ≤0.001: significant.

To understand the role of AIRE in selecting 2P24 skin-reactive Treg cells, we generated BM chimeras by reconstituting Aire^−/−^ and Aire^+^ recipients with 2P24 BM cells. 2P24 Treg generation was dramatically reduced in Aire^−/−^ recipients, by about tenfold (Fig. 2a top panels). Interestingly, Treg selection for the A12 clone, which showed no signs of autoimmunity on a Foxp3^sfy^ background, turned out to be independent of AIRE (Fig. 2a middle panels).

To confirm the role of AIRE in 2P24 Treg cell generation, we crossed 2P24 with Aire^−/−^ mice. 2P24 Aire^−/−^ mice had a greatly reduced (∼10-fold) Treg population compared with Aire^+^ 2P24 mice (Fig. 2a bottom panels). Interestingly, there were no signs of clonal deletion, as the total CD4 SP cells were not reduced in 2P24 Aire^+^ compared with Aire^−/−^ mice (Supplementary Fig. 4a). The decrease of Treg cells in Aire^−/−^ mice was already present at the Treg progenitor stage (CD4SP Foxp3^−^CD25^+^) (Supplementary Fig. 4b). Strikingly, despite the large tTreg reduction, 2P24 Aire^−/−^ mice remained healthy, without the skin autoimmune disease that afflicted 2P24 Foxp3^sfy^ mice (Supplementary Fig. 5a). In both 2P24 Aire^+^ and 2P24 Aire^−/−^ mice, the frequency of Treg cells in peripheral lymphoid organs was increased compared with the thymus, but the number and frequency of Treg cells in 2P24 Aire^−/−^ mice continued to be remarkably lower than in Aire^+^ mice (Supplementary Fig. 5b). These peripheral Treg cells were expanded thymic tTreg and not peripherally generated Treg cells (pTreg), as indicated by the earlier pTreg induction experiments carried out in 2P24 Aire^+^ mice^9^. We confirmed those findings by showing that the Treg cells expressed high levels of Nrp1 (refs 16, 17 and Supplementary Fig. 5c).

Although the lack of disease in 2P24 Aire^−/−^ mice suggested the possibility that the few remaining AIRE-independent Treg cells were sufficient to control skin autoimmune disease, it was also possible that the effector 2P24 cells in 2P24 Aire^−/−^ mice were defective in their capacity to induce skin disease. To study the potential influence of AIRE on 2P24 effector T cells, we generated four groups of BM chimeras, transferring 2P24 Foxp3^wt^ or Foxp3^sfy^ BM cells into Aire^−/−^ or Aire^+^ recipients. All recipients were Rag1^−/−^ to preclude the effect of recipient Treg cells. If 2P24 effector cells were defective in Aire^−/−^ conditions, 2P24 Foxp3^sfy^ donor-derived cells would not cause disease in Aire^−/−^ recipients. However, 2P24 Foxp3^sfy^ BM cells caused disease in both Aire^+^ and Aire^−/−^ mice (Fig. 2b). Thus, although AIRE is important for 2P24 Treg selection, AIRE-independent mechanisms give rise to a small population of Treg cells, which is sufficient to protect the mice under steady state.

We also tested whether 2P24 Treg cells were generated perinatally in an AIRE-dependent manner^18^, as the 2P24 TCR recognizes an antigen present in the adult skin. Indeed, from the earliest time point at P6, we could see a remarkable deficit in 2P24 Treg cell generation in Aire^−/−^ mice compared with Aire^+^ mice (Supplementary Fig. 6).

### *Bona fide* AIRE-independent 2P24 thymic Treg generation

**Figure 3:**
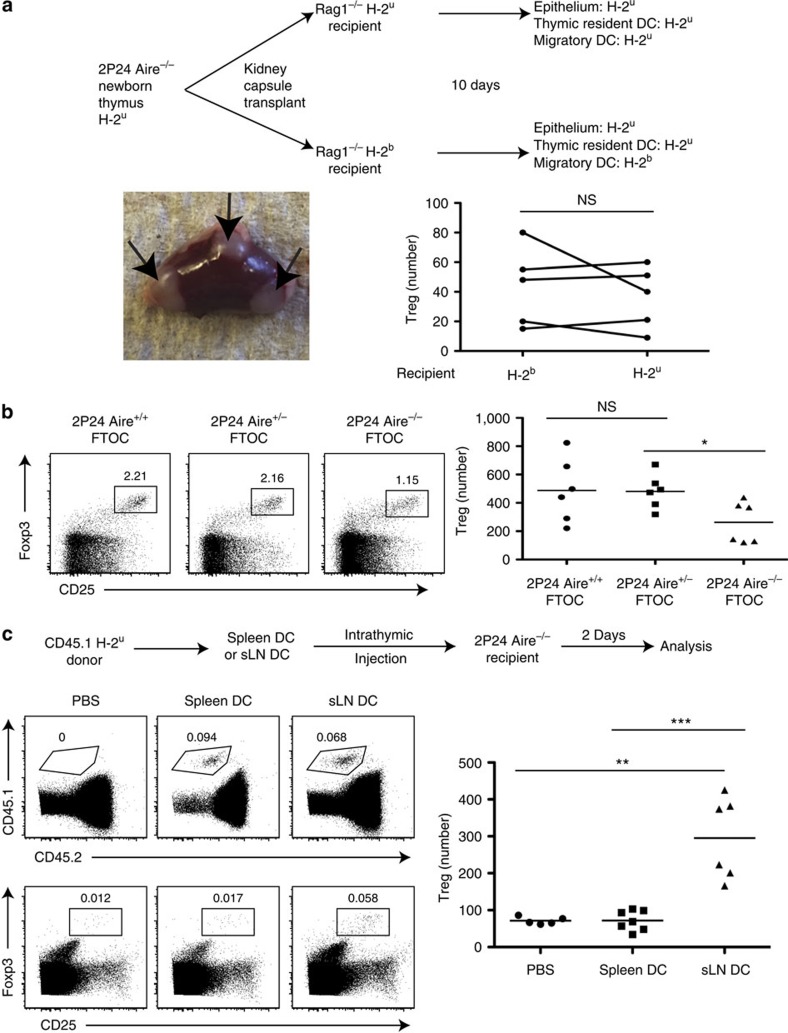
*Bona fide* AIRE-independent 2P24 thymic Treg generation. (**a**) Top: Scheme of kidney capsule transplantation experiments. Bottom left panel: picture of a kidney with three separate thymic lobes. Bottom right panel: Treg cell numbers per total thymic lobes. Results are representative of three independent experiments. (**b**) FTOC. E16 2P24 thymi (Aire^+/+^, Aire^+/−^ and Aire^−/−^) were cultured in tranwell plates for 7 days. Left three panels: representative staining of gated CD4SP cells. Right panel: Treg cell numbers per total cultured thymic lobes. Results are summary of two independent experiments. (**c**) Top: scheme of intrathymic injections; 0.1 million CD45.1 DC from the spleen or skin-draining LN (sLN) were injected into CD45.2 thymi. Middle row: recovery of injected cells, as shown by CD45.1 staining of total live cells. Bottom row: Treg staining gated on recipient CD45.2^+^CD4SP cells. Right panel: Treg cell numbers per total thymi. Results are summary of three independent experiments. Statistics in **a** were performed with paired Student's *t*-test and statistics in **b**,**c** were performed with unpaired Student's *t*-test. Error bars represent s.e.m. *P*-values >0.05: nonsignificant (NS); *P-values ≤0.05: significant; **P-values ≤0.01: significant; **P-values ≤0.001: significant.

Given the disease-protecting capacity of AIRE-independent 2P24 Treg cells, effective despite their small number, we studied the AIRE-independent induction of these cells. Theoretically, APC from the skin could migrate into the thymus and induce 2P24 tTreg generation. Indeed, the steady-state thymus contains a population of dendritic cells (DC) from the periphery, characterized as Sirpα^+^CD8α^−^CD11c^+^ (ref. 19), as well as migratory plasmacytoid DC. It has been shown that DC from fluorescein isothiocyanate-painted skin could migrate into the thymus^20^. Peripheral DCs were also shown to capture blood-borne antigens and carry them into the thymus, affecting negative selection^21^. To determine the impact of peripheral circulating DC on 2P24 thymic Treg selection, we used a thymus grafting technique into the kidney capsule, once again taking advantage of the strict H-2^u^ restriction of the 2P24 TCR. We grafted P1 neonatal thymic lobes, which lack migratory DC, from 2P24 Aire^−/−^ H-2^u^ mice under the kidney capsule of recipient H-2^b^ or H-2^u^ Rag1^−/−^ mice. We grafted lobes in a paired manner, that is, one thymic lobe was grafted into an H-2^b^ recipient, while the other lobe of the same thymus was grafted into an H-2^u^ recipient. It is known that peripheral DC from recipient mice migrate into the thymic grafts via blood^22^. Ten days after surgery, thymic implants were filled with migratory DC of recipient origin, whereas the epithelial cells remained H-2^u^, as expected. As 10 days is too short a time for the development of thymic resident DC from recipient BM cells, all resident Sirpα^−^CD8α^+^CD11c^+^ DC remained of graft origin (Supplementary Fig. 7). There was no difference in Treg selection between H-2^u^ and H-2^b^ recipient mice (Fig. 3a). Therefore, the contribution of peripheral DC to 2P24 thymic Treg selection appears to be minimal.

Another possibility is that these Treg cells could be peripherally developed pTreg that migrated into the thymus from the periphery. To determine whether there was thymic generation of 2P24 Treg cells in the absence of AIRE, and without the possibility of peripheral migration of pTreg, we employed fetal thymus organ culture (FTOC). We observed Treg generation in the 2P24 Aire^−/−^ FTOC (Fig. 3b), which demonstrated that migration of mature pTreg cells from the periphery was not necessary to generate Treg cells in Aire^−/−^ thymi, and once again confirmed that migratory DC are not necessary.

Despite the lack of evidence for a physiologic role of migratory DC in 2P24 tTreg generation, we tested whether such peripheral DC-induced 2P24 Treg development could be forced. We purified H-2^u^ CD11c^+^ cells from either the spleen or the skin-draining lymph nodes and transferred them intrathymically into 2P24 Aire^−/−^ mice. Mice receiving DC from skin-draining lymph nodes had, on average, threefold more 2P24 Treg cells compared with mice receiving splenic DC or PBS (Fig. 3c). Regardless of the physiologic role of peripheral APC in the generation of 2P24 Treg cells, this observation suggests an intervention whereby skin-specific Treg cells could be specifically augmented, a possibility further explored below.

### 2P24 Treg selection requires haematopoietic APC

AIRE-expressing mTEC are able to present antigens and select Treg cells directly^23^,^24^,^25^. mTEC could also transfer antigens to DC^26^ for re-presentation and thymic DC have also been found to be involved in Treg selection^22^,^27^,^28^. Studies with OVA-specific OT-II T cells in thymus organ cultures found that almost all types of APC could help select Treg cells when loaded with OVA^29^. Transduction of a large number of TCR from Treg cells into Rag1^−/−^ thymocytes identified clones that were either selected by haematopoietic APC or mTEC^30^.

**Figure 4:**
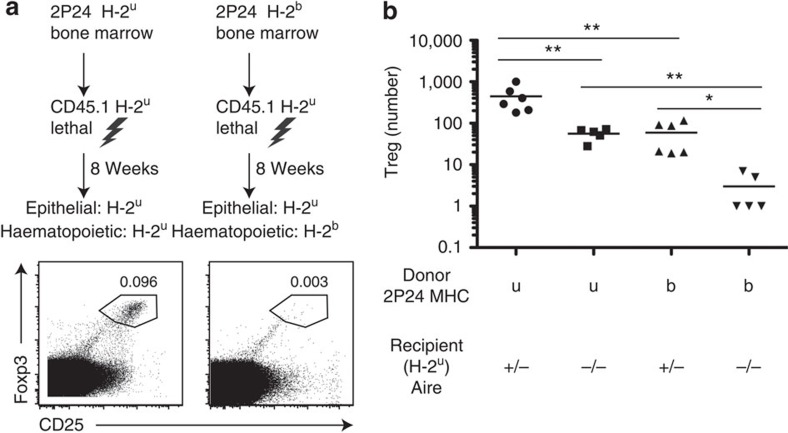
2P24 Treg selection requires haematopoietic APC. (**a**) Scheme of mismatched BM chimeras. Mice in both groups were treated with anti-NK1.1 antibody for the first 3 weeks after transfer. Representative thymus staining was gated on CD4SP cells. (**b**) BM chimeras were created in the same way as in **a**, with the inclusion of Aire^−/−^ recipient groups. Summary of Treg cell numbers in the BM chimeras. Results are the summary of three independent experiments. Statistics were performed with unpaired Student's *t*-test. Error bars represent s.e.m. *P*-values >0.05: nonsignificant (NS); *P-values ≤0.05: significant; **P-values ≤0.01: significant.

To explore which APC(s) induce the development of 2P24 Treg cells in the thymus, we generated BM chimeras by reconstituting lethally irradiated H-2^u^ mice with 2P24 H-2^u^ or 2P24 H-2^b^ BM cells. In both groups the epithelial cells expressed the selecting H-2^u^ MHC, whereas the haematopoietic APC were either H-2^u^ or H-2^b^. There was a dramatic reduction of Treg cells when haematopoietic APC expressed the non-selecting H-2^b^ MHC, despite the fact that AIRE-expressing mTEC cells and cortical epithelial cells expressed the selecting H-2^u^ molecules (Fig. 4a). Furthermore, when we carried out the same experiment in AIRE^−/−^ recipient mice, it became clear that efficient 2P24 Treg development required both AIRE and haematopoietic-derived APC (Fig. 4b, note the log scale). Thus, both AIRE-dependent and AIRE-independent Treg development require haematopoietic APC, to effectively generate 2P24 Treg cells.

### Increasing mTEC dramatically increases 2P24 Treg selection

**Figure 5:**
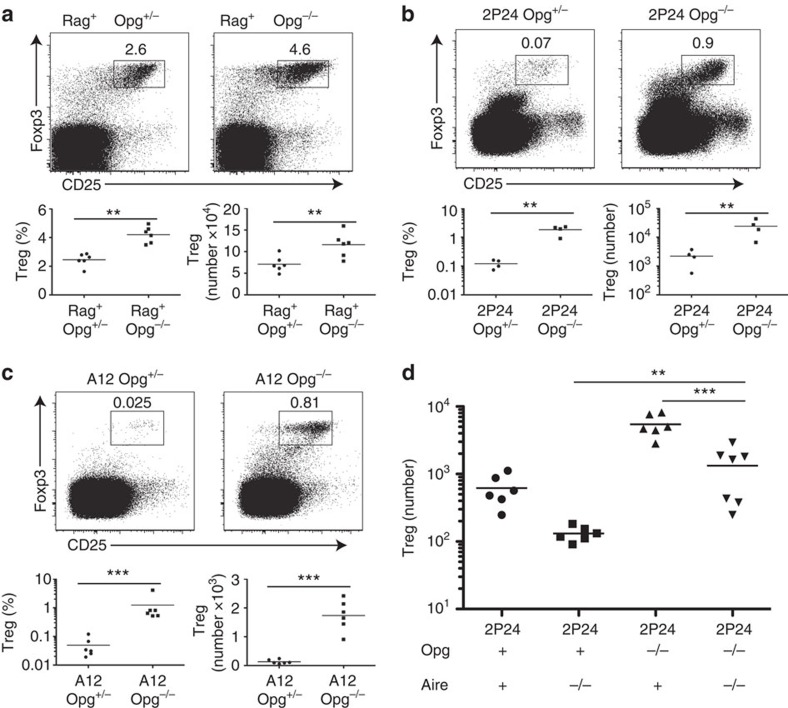
Increasing mTEC dramatically increases Treg selection. (**a**) Polyclonal Opg^+/−^ and Opg^−/−^mice. Top two panels: representative flow cytometry of CD4SP gated cells. Bottom two panels: Treg cell percentages and numbers. Results are the summary of two independent experiments. (**b**) 2P24 Opg^+/−^ and Opg^−/−^mice. Top two panels: representative flow cytometry of CD4SP gated cells. Bottom two panels: Treg cell percentages and numbers. Results are the summary of two independent experiments. (**c**) A12 Opg^+/−^ and Opg^−/−^mice. Top two panels: representative flow cytometry of CD4SP gated cells. Bottom two panels: Treg cell percentages and numbers. Results are the summary of two independent experiments. (**d**) Treg cell numbers in 2P24 Opg^+^ Aire^+^, Opg^+^ Aire^−/−^, Opg^−/−^ Aire^+^ and Opg^−/−^ Aire^−/−^ mice. Results are the summary of three independent experiments. Statistics were performed with unpaired Student's *t*-test. Error bars represent s.e.m. *P*-values >0.05: nonsignificant (NS); **P-values ≤0.01: significant; ***P-values ≤0.001: significant.

Given that 2P24 Treg generation in the thymus requires, for the most part, AIRE-expressing mTEC and re-presentation by haematopoietic APC, we sought to test the small niche hypothesis^9^,^11^, which predicts that the small but relatively fixed number of clonal tTreg cells generated in 2P24 thymi reflects the limited availability of antigen required to drive Treg generation. Studies with different genetic models showed that in polyclonal T-cell systems, increased thymic medulla led to higher Treg numbers, whereas decreased thymic medulla resulted in reduced Treg numbers^31^,^32^,^33^. Osteoprotegerin (OPG, Tnfrsf11b) is a soluble decoy factor, as it competes with RANK for the binding of TRANCE/RANKL/Tnfsf11. OPG limits thymic medulla development and, expectedly, Opg^−/−^ mice have enlarged thymic medulla^34^,^35^. Opg^−/−^ mice with polyclonal T-cell repertoires have been reported to have a modest increase in thymic Treg cells^33^,^34^, an observation that we confirmed (Fig. 5a). There is no agreement as to whether the modest Treg increase is due to *bona fide* thymic generation of Treg cells in the thymus^33^,^34^. However, observations with polyclonal T cells can mask TCR repertoire adjustments. We thus studied the role of OPG in 2P24 Treg cells, which have a fixed T-cell receptor, by crossing 2P24 with Opg^−/−^ mice. Remarkably, 2P24 Opg^−/−^ mice had about tenfold higher numbers of thymic Treg cells compared with 2P24 Opg^+/−^ mice (Fig. 5b). Treg development in the thymus transits from early precursors (CD4SP Foxp3^−^CD25^+^)^36^,^37^, via immature CD4SP stage (CD69^+^CD62L^−^) to the more mature CD4SP stage (CD69^−^CD62L^+^)^38^. The absence of Opg resulted in a clear increase of Treg cells at all developmental stages, from precursors to mature SP cells, indicating that Opg affected *bona fide* thymic Treg generation in 2P24 mice (Supplementary Fig. 8a,b). Interestingly, A12 Opg^−/−^ mice, whose Treg selection is largely AIRE independent, also had five- to tenfold more Treg cells than A12 Opg^+/−^ mice (Fig. 5c).

We next sought to examine the changes in epithelial and DC populations in 2P24 Opg^−/−^ thymi, as it was possible that dramatic changes in the mTEC compartment could be followed by changes in the haematopoietic APC compartment. As expected, thymi from Opg^−/−^ mice had a greatly expanded mTEC population, including the more mature mTEC, which are MHCII^hi^CD80^hi^ (Supplementary Fig. 9a). In contrast to mTEC, DC were unaffected in number and phenotype (MHCII, CD80 and CD86 expression levels; Supplementary Fig. 9b).

To determine the selectivity of OPG deficiency to AIRE-dependent and AIRE-independent 2P24 Treg selection in the thymus, we analysed 2P24 Opg^−/−^ Aire^−/−^ mice. Eliminating AIRE expression led to a large reduction in Treg cell number compared with 2P24 Opg^−/−^ mice (Fig. 5d). Thus, the dramatic increase in the number of Treg cells in 2P24 Opg^−/−^ mice was largely accounted for the increase in AIRE-dependent antigens. Remarkably, the number of Treg cells in Opg^−/−^ Aire^−/−^ mice was still much higher than the Treg number found in 2P24 Aire^−/−^ mice (Fig. 5d). As there is an AIRE-independent generation of 2P24 Treg cells (Fig. 3), we concluded that the increased mTEC population in Opg^−/−^ mice comprises the expansion of mTEC expressing both AIRE-dependent and AIRE-independent antigens.

We next tested the effect of TRANCE addition on Treg cell generation in thymic organ cultures (FTOC). We treated 2P24 FTOC with TRANCE/RANKL. TRANCE/RANKL had been shown to enhance the development of mTEC, including AIRE-expressing mTEC^35^,^39^. In agreement with the data obtained with OPG deficiency, TRANCE addition increased 2P24 Treg cell generation; however, it was at the expense of total CD4SP cells (Supplementary Fig. 10a). Nonetheless, TRANCE had no effect on Treg induction in FTOC with T cells expressing Tconv TCR (Supplementary Fig. 10b), supporting the model whereby a ‘correct' TCR is needed for tTreg generation. TRANCE administration increased the proportion of mTEC cells, in particular the more mature MHCII^hi^CD80^hi^ population (Supplementary Fig. 10c). It is likely to be that by increasing thymic mTEC with TRANCE, we increased the amount of high-affinity ligands for the 2P24 clone, which led to increased Treg selection and negative selection. Consistent with these observations, crossing 2P24 with TRANCE^−/−^ mice resulted in a major reduction of Treg cell numbers (Supplementary Fig. 10d).

### Skin extracts injected intrathymically boost 2P24 Tregs

Although the 2P24 Opg^−/−^ data was informative regarding the role of mTEC expansion in Treg selection, it left us one step removed from proving that antigen availability was the limiting factor for Treg cell selection. Fortunately, 2P24 TCR skin reactivity allowed us to directly test this hypothesis.

**Figure 6:**
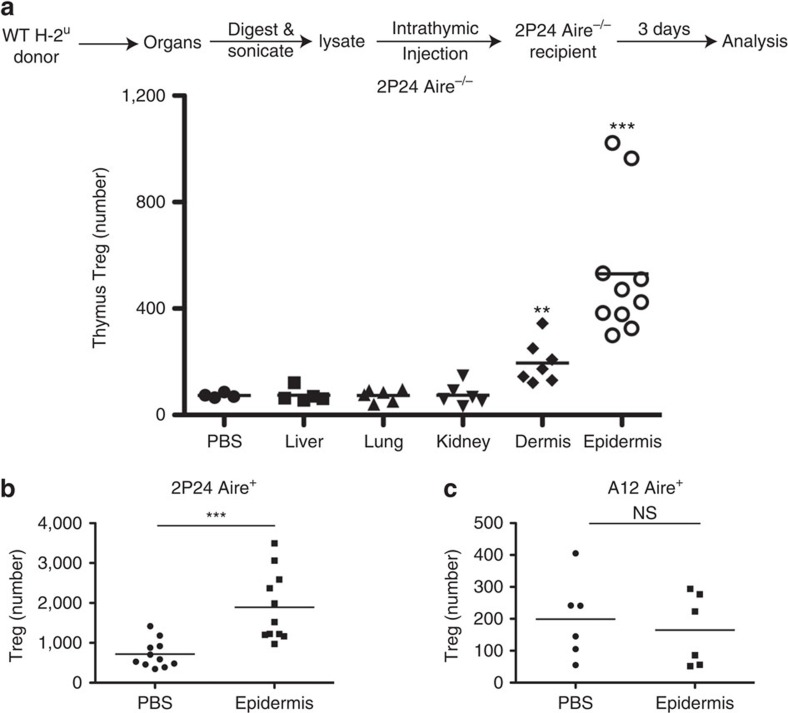
Skin extracts injected intrathymically boost 2P24 Tregs. (**a**) Single cells prepared from the indicated organs of H-2^u^ WT mice were sonicated for 5 min. The total extracts (including the soluble part and the debris) were intrathymically injected into 2P24 Aire^−/−^ mice. Each mouse received an equivalent of one million cells. Three days later, the recipients' thymi were analysed by flow cytometry. Shown is the Treg numbers per total thymi. Results are the summary of five independent experiments. (**b**) Epidermal extracts were injected intrathymically into 2P24 Aire^+^ mice in the same way as in **a**. Shown is Treg numbers per total thymi 3 days after the injection. Results are the summary of three independent experiments. (**c**) The same epidermal extracts were injected intrathymically into A12 Aire^+^ mice in the same way as in **a**. Shown is Treg numbers per total thymi 3 days after the injection. Results are the summary of two independent experiments. Statistics were performed with unpaired Student's *t*-test. Error bars represent s.e.m. *P*-values >0.05: nonsignificant (NS); *P-values ≤0.05: significant; **P-values ≤0.01: significant; ***P-values ≤0.001: significant.

We prepared cell lysates from the skin and from other organs of wild-type (WT) mice, and injected the lysates intrathymically into 2P24 Aire^−/−^ mice. We found that epidermal lysates could indeed increase thymic 2P24 Treg cell number by an average of tenfold (Fig. 6a). The increase was observed in both immature and mature CD4SP Treg cells (Supplementary Fig. 11a), suggesting *bona fide* thymic Treg generation. However, there was no increase of Treg precursors (Supplementary Fig. 11b). This is probably due to the fact that we only analysed the results 3 days after intrathymic injection of epidermis extracts; therefore, only one wave of Treg development was probably affected. After 3 days, most precursors probably had differentiated into the immature Treg stage. Thymic injection of skin extracts could have also caused deletion of conventional 2P24 T cells. However, the Treg cell increase that we observe was not accompanied by any reduction in the number of Tconv cells (Supplementary Fig. 11c).

As expected from the pathology of 2P24 Foxp3^sfy^ mice, epidermal lysates were more effective than dermal lysates, whereas lysates from other organs had no impact on thymic 2P24 Treg cell generation.

Epidermal extracts were also effective at boosting almost threefold Treg numbers in 2P24 AIRE^+^ mice, whereas the same extracts did not affect Treg cell numbers in A12 mice, indicating that the epidermal extracts do not contain a universal Treg-inducing factor and thus the inducting effect is TCR specific (Fig. 6b,c).

We could therefore boost Treg selection of a Treg clone of which the tissue reactivity was known, but the actual epitope/protein was not. This opens a path for therapeutic interventions directed at raising specific tTreg populations.

## Discussion

Studies on TCR transgenic mice made from Treg TCR showed the selection of Treg cells, albeit reaching a ceiling at a low total number^9^,^10^,^11^. Given that the WT thymus supports the development of a higher number of Treg cells, the limiting factor in the case of monospecific Treg developing in TCR transgenic:WT mixed chimeras could not be the availability of growth factors. We and others put forward a possible explanation for this low ceiling, whereby Treg cells could only receive inductive signals at specific thymic niches, which were rare and different for each TCR. Niches would contain TCR ligands (agonists), which induce or consolidate the Foxp3-dependent pathway^9^,^11^,^40^. However, little was known about the ligands that induce ‘real' Treg cells.

Before these experiments with Treg TCR, much had been learned in the field by taking TCR from Tconv cells and altering the cognate antigen delivery to obtain Treg selection. These pioneering experiments led to the conclusion that thymic T cells were selected into the Treg compartment if they encountered moderate/high avidity ligands^6^,^7^,^8^,^40^,^41^,^42^.

The issue of self-reactivity of tTreg cells has been highly debated. It has been shown that four of ten TCR transgenic T cells retrovirally transduced with TCRα chains from CD4^+^CD25^+^ cells rapidly expanded in lymphopenic hosts, but the others proliferated poorly or not at all. No TCRα chain from CD4^+^CD25^−^ cells (0/10) conferred high proliferative capacity to host T cells^43^. It remains unclear whether more than half of the TCR from Treg cells are much less self reactive, or the proliferation assay did not detect their autoreactivity. Using pooled TCRα chains from Treg cells, marked weight loss was observed in recipient mice, whereas no such weight loss was observed when pooled TCR from Tconv cells were used^43^. However, it is unclear to what extent the weight loss reflects T-cell reactivity against self or non-self antigens^44^. Mice from a prostate-specific Treg TCR transgenic line display infiltration in the prostate, showing clear autoreactivity^45^. However, the preferential reactivity of Treg TCR to self antigens has been questioned altogether after testing the reactivity of hundreds of hybridomas expressing Treg and Tconv TCR from a mini TCRα locus^44^.

In this study, we determined that one TCR obtained from a FOXP3^+^ Treg cell (2P24) recognizes an antigen present in the skin. It is thus an autoreactive TCR. When the FOXP3 programme was abolished by crossing 2P24 with scurfy mice, all mice developed severe skin inflammation, which included focal hair loss. Autoimmune alopecia is a frequent autoimmune disease in men, reaching 2% incidence^46^,^47^. Despite the focal hair loss observed in 2P24 Foxp3^sfy^ mice, it is unclear to what degree the pathology in these mice reproduces human autoimmune alopecia, or other known animal models of the disease. We have thus developed an experimental model in which T cells expressing the TCR derived from a Treg cell induce, with high penetrance, an organ-specific disease in the absence of Foxp3. In the process, we have proven, beyond doubt, the pathogenic autoreactivity potential of the naturally occurring 2P24 Treg TCR.

Skin-resident Treg cells have been shown to be indispensable for maintaining the immune homeostasis in the skin^48^. Interestingly, TCR transgenic mice expressing a skin-reactive TCR originally selected from Foxp3-deficient mice (SFZ70) were able to spontaneously generate thymic FOXP3^+^ Treg cells in large numbers^49^. SFZ70 transgenic Rag1^−/−^ mice developed a fulminant disease when crossed to scurfy, with moribund mice by 24 days of age; this death occurs faster than the polyclonal scurfy mice^49^. The original SFZ70 TCR could have been a Treg TCR, but it is difficult to determine due to its scurfy origin. It is also possible that the original SFZ70 cells represented one of these Tconv cells that are prevented from activation and expansion by Treg cells^50^.

The importance of thymus epithelium in Treg selection has been known for a long time^51^. More recently, it has been shown that mTEC are critical for this process^23^,^25^, although cortical thymic epithelial cells (cTEC) were also postulated to be able to select Treg cells^52^. A fraction of mTEC cells express AIRE, whose product controls the expression of a large number of TRAs^13^,^15^,^53^,^54^,^55^.

For several years, AIRE involvement in Treg cell generation was considered minimal or non-existent, as AIRE-deficient mice had no significant difference in the number of Treg cells and TCR repertoire compared with WT mice^13^,^56^,^57^,^58^,^59^. However, studies with clonal Treg cells have indicated a strong AIRE involvement. Two prostate-specific Treg clones were almost completely dependent on AIRE for their selection, even in female mice^45^. A study of 11 Treg TCRα chains transduced into RAG^−/−^ thymocytes and injected into thymi of Aire^+^ or Aire^−/−^ recipients showed that Treg generation for 6 of the 11 clones had substantial AIRE dependence, whereas the remaining 5 clones were AIRE independent^30^. In this manuscript, we studied Treg TCR transgenic mice expressing two different TCR and found that one of them, 2P24, had a strong, although not absolute, AIRE dependence, whereas the other TCR, A12, developed into the Treg programme regardless of AIRE. In recent times, the involvement of AIRE in Treg generation has been shown to be restricted to perinatal Treg cells^18^. The AIRE-dependent 2P24 TCR was, indeed, already present at the perinatal period. Its junctional Vα-Jα TCR sequence has, however, 3N nucleotides; N nucleotide addition is known to be rare at the perinatal period^60^. It is therefore possible that the original 2P24 Treg was not generated perinatally.

In addition to mTEC, AIRE expression was recently described on thymic B cells^61^. However, given the fact that we used TCR transgenic Rag1^−/−^ mice, B cells are irrelevant here.

Our FTOC studies using 2P24 Aire^−/−^ and Aire^+^ thymi showed a less dramatic difference than adult mice or BM chimeras with adult recipient mice. One possible explanation is that the AIRE^+^ medullary epithelial cells have not developed at E16 when we start the FTOC and do not develop well in the FTOC, blurring the differences between Aire^+^ and Aire^−/−^ FTOC. In any case, we showed that there is some *bona fide* 2P24 thymic Treg generation in the absence of AIRE. Taken together with the Opg^−/−^Aire^−/−^ data, it is likely to be that mTEC express a low level of 2P24 ligands in the absence of AIRE.

It is interesting that Treg generation of the A12 TCR was AIRE independent, suggesting that A12 does not recognize a TRA. Accordingly, we did not found any signs of inflammation or peripheral tissue damage in A12 scurfy mice. However, OPG deficiency, which increased mTEC, also increased A12 Treg selection. Thus, similar to the 2P24 case, mTEC appears to express the ligand recognized by A12 T cells.

For the 2P24 T cells, the predominantly AIRE-dependent antigen requires re-presentation by haematopoietic APC, most probably DC. The paradox is that mTEC, in particular AIRE-expressing mTEC, are competent to present antigen and directly induce Treg cells^23^,^30^,^62^. Re-presentation of mTEC antigens by thymic DC has been known to occur^26^,^30^,^63^,^64^, but the reasons for its requirement for some Treg cells are unclear. Re-presentation by DC may reach what for some Treg TCR is the adequate level of antigen to induce Treg generation^65^.

The dramatic increase of 2P24 and A12 Treg in Opg^−/−^ mice provided further support to the limiting niche hypothesis and also to the instructive role of TCR in Treg cell selection. The presence of Treg cells in Treg TCR transgenic mice, as opposed to their absence in Tconv TCR transgenic mice, suggested that having the ‘correct' Treg TCR was a prerequisite for Treg selection. The reason why the majority of T cells in Treg TCR transgenic mice did not become Treg in spite of expressing the ‘correct' TCR is likely to be the limited availability of high-affinity ligands in the thymus. In the present work we showed that by increasing the high-affinity ligands through increasing mTEC, we could greatly increase Treg cell selection. It should be noted that Treg selection also requires TCR-independent factors such as cytokines (interleukin (IL)-2 and IL-15), which are believed to be the major limiting factor for Treg selection in polyclonal systems^36^,^66^,^67^. This explains why we only observed a modest (∼2 fold) increase of Treg cells in the polyclonal Opg^−/−^ mice, as further increase of Treg cells in this system may have been prevented by the limiting cytokines, whereas the Treg frequency in 2P24 and A12 mice was too low to be limited by cytokines.

In recent times, a paper was published that challenged the limiting niche hypothesis of Treg T-cell selection^68^. In our view, the investigators used a constricted experimental system, not only eliminating MHC–peptide diversity but also limiting the TCR diversity to one TCRβ chain, and within the TCRα chain only one Vα and only two Jα. The simplification of the system might have been pushed beyond the point in which it can freely adapt. Even using this experimental system, the authors obtained quantitative changes in TCRα frequencies from Treg and Tconv cells. The limited niche hypothesis proposes that positive selection takes place normally in T cells that will become Treg cells; this is triggered by a number of low avidity interactions. Subsequently, a more restrictive interaction is required to trigger the Foxp3 programme. Any T cell exceeding the capacity of the niche would remain a positively selected Tconv cell; however, as shown in 2P24 mice, these Tconv cells would not be pathogenic because of Treg cells with the same specificity that were generated in the niche. If the niche were empty, then Treg development would take place.

Our work showed that intrathymic delivery of an epidermis lysate increased 2P24 Treg generation. This was highly specific as lysate from other organs failed to induce more 2P24 Treg cells and the epidermis lysate failed to increase A12 Treg cells. Combined with the data from OPG-deficient mice, this experiment further supports the hypothesis that 2P24 Treg selection is limited by the amount of high-affinity ligands in the thymus. More practically, this assay indicates a way to increase antigen-specific Treg cells from the thymus without knowing the exact antigen (epitope or even protein). This could be clinically relevant in autoimmune settings where a certain organ is being targeted but the exact antigen(s) remains to be defined. Altogether, our work clarifies some of the intricacies of Treg cell selection in the thymus and opens the way for future studies and possible clinical applications.

## Methods

### Mice

Aire^−/−^ mice and Opg^−/−^ mice were obtained from The Jackson Laboratory on a C57BL/6 background. TRANCE^−/−^ mice were previously reported^69^. All these mouse strains were backcrossed onto the C57BL/10.PL background for at least five generations. CD45.1 Aire^−/−^ mice were generated through breeding Aire^−/−^ mice with CD45.1 C57BL/10.PL mice. 2P24 and A12 Treg TCR transgenic mice have been previously described^9^. 2P24 and A12 mice are always Rag1^−/−^ unless otherwise stated. Six- to 8-week-old mice of both sexes were used for experiments.

Animals were housed at the NYU Medical Center Skirball Animal Facility under SPF conditions. All procedures were approved by New York University School of Medicine Institutional Animal Care and Use Committee.

### Antibodies and reagents

The following conjugated antibodies were used: CD4 (RM4–5), CD8α (53–6.7), CD44 (IM–7), CD62L (MEL–14), CD3 (17A2), CD25 (3C7), CD69 (H1.2F3), CD45 (30–F11), CD45.1 (A20), CD45.2 (104), Sirpα (P84), CD11c (N418), CD11b (M1/70), CD80 (16–10A1), CD86 (GL–1), I-A/I-E (M5/114.15.2), interferon-γ (XMG1.2), IL–17A (TC11–18H10.1), EpCam (G8.8), Ly51 (6C3), Foxp3 (FJK–16s), RT1B (OX–6) and I-A^b^ (AF6–120.1). All the above antibodies were from Biolegend or eBioscience. Biotinylated rNrp1 antibody was from R&D Systems. Ulex Europaeus Agglutinin I was from Vector Laboratories, Inc.. Anti-NK1.1 antibody (Clone PK136) was from BioXCell. Depending on the antibody, a range of dilutions between 1:200 and 1:1,000 was used.

Anti-CD8 microbeads, anti-CD4 microbeads, anti-PE microbeads, anti-CD11c microbeads, LS columns and LD columns were from Miltenyi Biotech.

### Flow cytometry

Cells were stained with antibodies following standard procedure. Intracellular staining of Foxp3 was performed using the eBioscience kit according to the manufacturer's instructions. For intracellular cytokine staining, cells were activated with phorbol myristate acetate/ionomycin for 2 h and then monensin (Golgi stop) for 2 h, and cells were then fixed/permeabilized and stained using the BD Biosciences kit according to the manufacturer's instructions.

For whole thymus analysis, single-cell suspensions were prepared from whole thymi and 95% of thymi were depleted of CD8 cells by labelling cells with anti-CD8 microbeads and passing through magnetic LD columns. Total sample of the CD8-depleted fractions and the 5% unmanipulated fractions were analysed.

Stained cells were analysed on a LSRII flow cytometer (BD) and data processed using FlowJo (Tree Star).

### Cell isolation from dermis and epidermis

Skin patches from mouse tails were digested with 0.25% trypsin overnight at 4 °C or 2 h at 37 °C. The epidermis and dermis were then separated. Epidermis was minced and further digested with 0.1% trypsin at 37 °C for 1 h. Dermis was minced and digested with 0.35% Collagenase I at 37 °C for 1 h. Digested dermis and epidermis were then passed through 70-μm cell strainers. In some experiments, lymphocytes were enriched using Percoll gradient centrifugation.

### Preparation of lysate from different organs

Lungs, livers and kidneys were minced and digested with 0.35% Collagenase I and 1% DNase I at 37 °C for 1 h. Cells were then passed through 70-μm cell strainers and counted. Fifty million cells were resuspended in 500 μl PBS and subjected to sonication (4 °C with water bath) for 10 min (30 s on and 30 s off, 10 cycle in total). The lysate was directly used for intra-thymic injection or *in vitro* cell culture experiments.

### Isolation of DC from the spleen and lymph nodes

The spleen and lymph nodes were minced and digested with Collagenase D and DNase I at 37 °C for 40 min. Cells were then passed through 70-μm cell strainers. Purification of DC was achieved by labelling cells with anti-CD11c microbeads and subsequent positive selection with LS columns.

### Thymic DC and epithelial cells

For thymic DC analysis, thymus was minced and digested with Collagenase D and DNase I at 37 °C for 40 min. Cells were then passed through 70-μm cell strainers and were ready to be used for staining.

For thymic epithelial cells analysis, the thymus was minced and digested with Dispase, Collagenase D and DNase I at 37 °C for 40 min. Cells were then passed through 70-μm cell strainers. Epithelial cells were enriched by PBS/52.7% Percoll/92.4% Percoll gradient centrifugation.

### Histology

Tissues were fixed in 10% neutral buffered formalin and embedded in paraffin. Five-micrometre sections were stained with haematoxylin and eosin.

### Bone marrow chimeras

Recipient mice were sublethally or lethally irradiated and reconstituted with BM cells from A12 or 2P24 mice (depleted of mature CD4 and CD8 cells). In some experiments involving mismatch transplant, the recipients were treated with anti-NK1.1 antibody 3 days before reconstitution and continued for 2 weeks after reconstitution. After 2 months, mice were killed, and thymi and peripheral lymphoid organs were removed for analysis.

### Ultrasound-guided intrathymic injections

Four- to 8-week-old mice were anaesthetized with 4% Isoflurane (Aerrane) in medical air and maintained under anaesthesia using a nose cone with 1.5% Isoflurane. Hair was removed from the thorax using with depilatory cream and animals were placed on a heat pad set at 37 °C during the injection procedure (∼2–3 min per mouse). Thymus was visualized with a 30-MHz 707B ultrasound probe (VisualSonics). Cells resuspended in PBS or cell lysate in PBS were injected (10 μl in each lobe) using a Hamilton syringe and a 30-gauge needle with the aid of a three-dimensional micromanipulator. Two to 3 days after the injection, the thymus (and, in some experiments, spleen) was harvested for analysis.

### Fetal thymic organ cultures

Thymic lobes were isolated from E16 embryos and cultured on transwell plates (Corning 3421). Complete RPMI with 20% fetal bovine serum was used for culture. FTOCs proceeded for 7–14 days and were subsequently analysed by flow cytometry.

### Kidney capsule transplants

Thymic lobes from 1-day-old donor mice (P1) were grafted under the kidney capsule of anaesthetized 8-week-old recipient mice using a procedure previously described^70^. Two to three thymic lobes were grafted in one kidney and the locations were recorded, to compare the thymic lobe from the same pair, which was grafted in another recipient. At specified times post grafting, grafted thymic lobes were recovered and processed individually. Thymic lobes were digested in Collagenase D and DNase I, and analysed by flow cytometry.

### Statistical analysis

Mean and s.e.m. values were calculated with GraphPad Prism (GraphPad Software). Unpaired or paired Student's *t*-tests were used to compare two variables, as indicated in each figure legend. *P*-values >0.05: nonsignificant; *P-values ≤0.05: significant; **P-values ≤0.01: significant; ***P-values ≤0.001: significant.

## Additional information

**How to cite this article:** Lin, J. *et al.* Increased generation of Foxp3^+^ regulatory T cells by manipulating antigen presentation in the thymus. *Nat. Commun.* 7:10562 doi: 10.1038/ncomms10562 (2016).

## Supplementary Material

Supplementary Information Supplementary Figures 1-11
