# Supplementary material for: Increased generation of Foxp3+ regulatory T cells by manipulating antigen presentation in the thymus
Source: Nat Commun. 2016 Feb 29;7:10562. doi: 10.1038/ncomms10562 (PMC4773449; doi:10.1038/ncomms10562)
Supplement: Supplementary Information — Supplementary Figures 1-11 [file ncomms10562-s1.pdf]

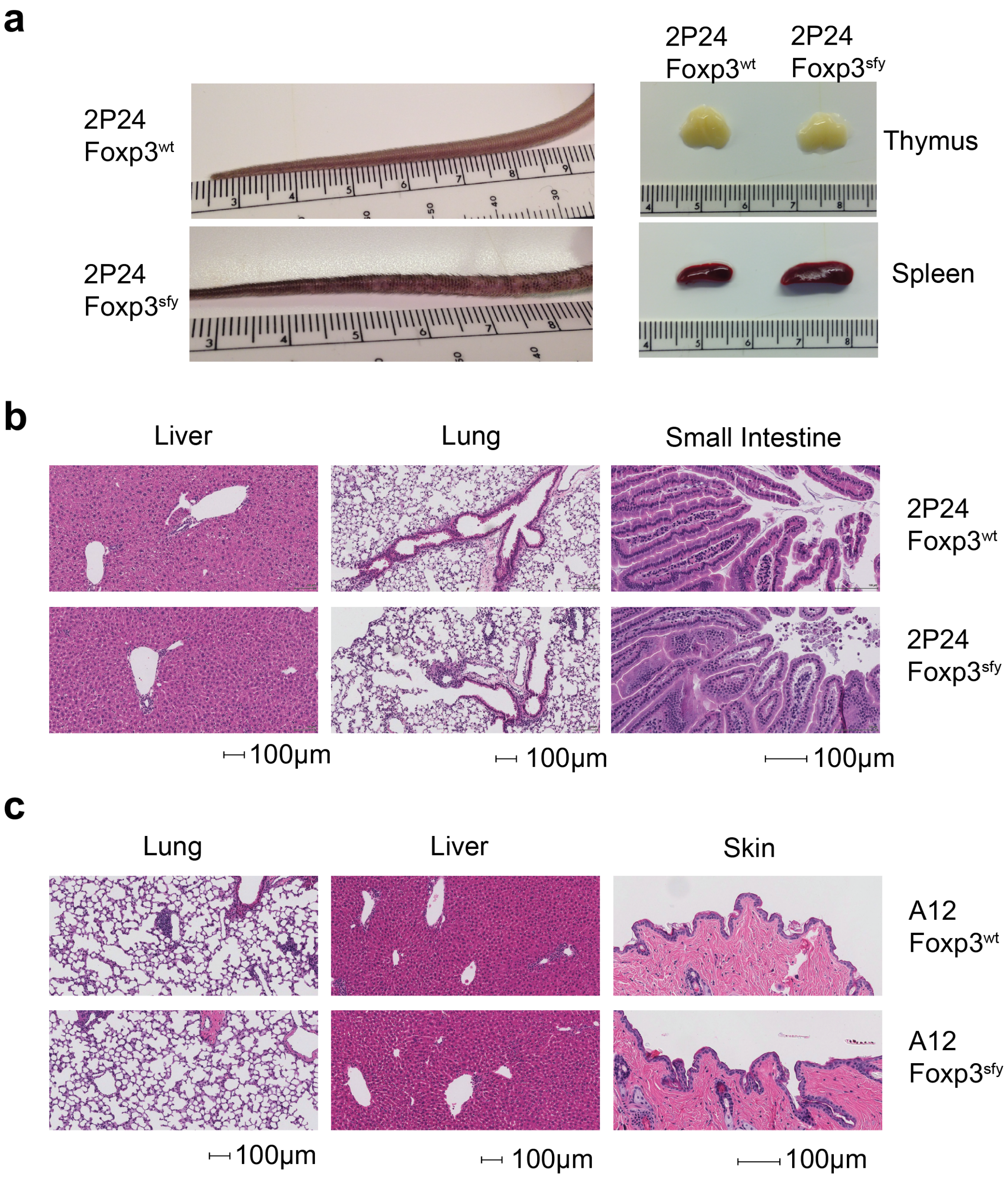

Supplementary Figure 1

Supplementary Figure 1. Pathology in 2P24 Foxp3^{sfy} mice is limited to the skin while ther is no pathology in A12 Foxp3^{sfy} mice

(a) Tail, thymus and spleen of 2 month-old 2P24 Foxp3^{wt} and 2P24 Foxp3^{sfy} mice.

(b) H&E staining of liver, lung and small intestine of 2 month-old 2P24 Foxp3^{wt} and 2P24 Foxp3^{sfy} mice.

(c) H&E staining of liver, lung and skin of 2 month-old A12 Foxp3^{wt} and A12 Foxp3^{sfy} mice.

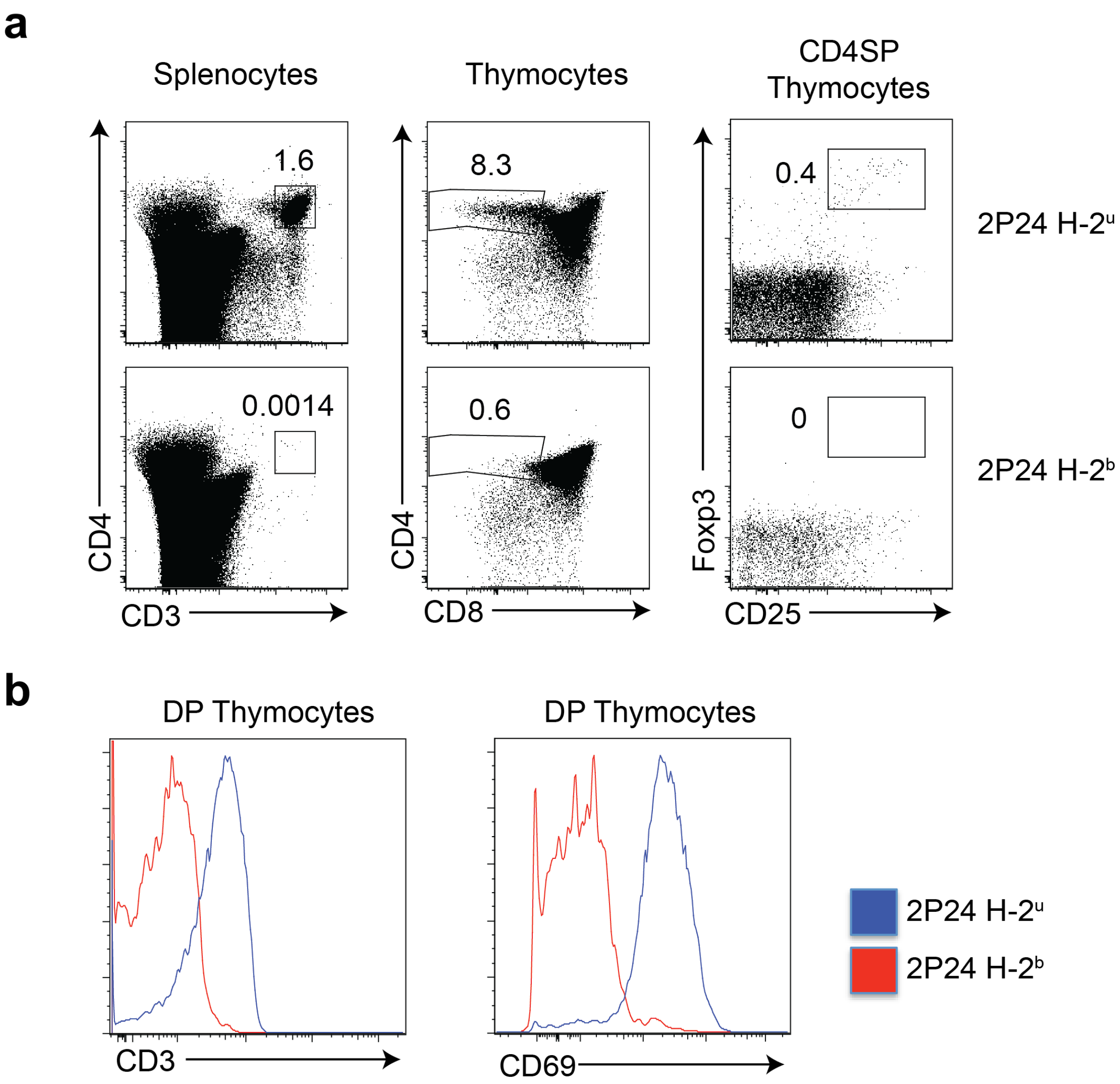

Supplementary Figure 2

Supplementary Figure 2. An MHC mismatch experimental system for the 2P24 Treg TCR

(a) Flow cytometry of spleen cells and thymocytes from 2P24 H-2^b and 2P24 H-2^u mice.

(b) CD3 and CD69 expression on DP thymocytes from 2P24 H-2^b and 2P24 H-2^u mice.

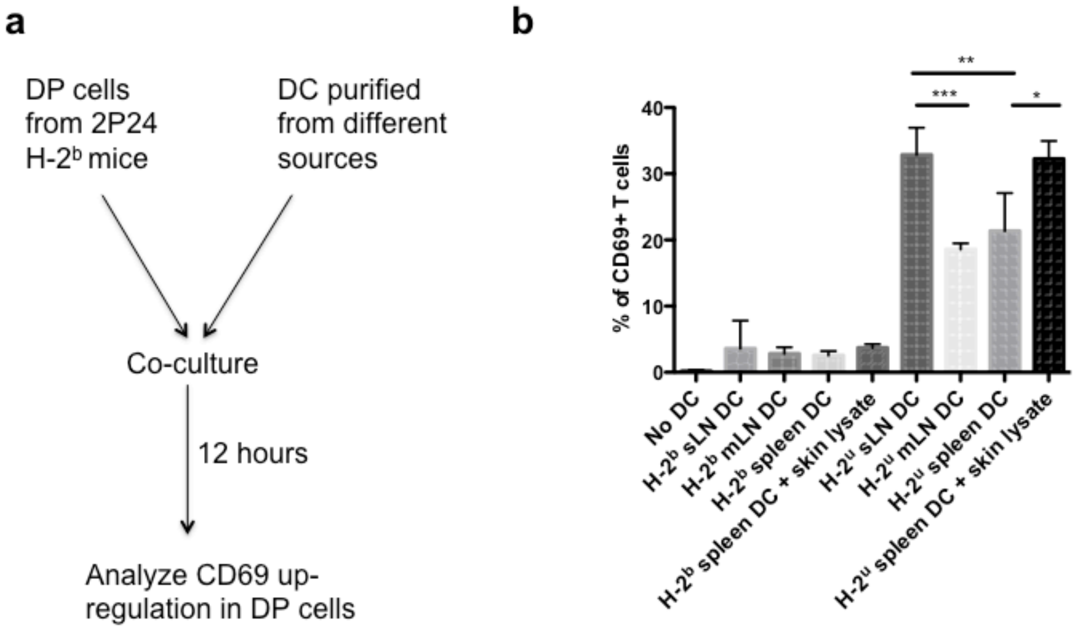

Supplementary Figure 3

Supplementary Figure 3. *In vitro* co-culture confirms the skin-reactivity of the 2P24 Treg TCR. DP thymocytes from 2P24 H-2^b mice were isolated and co-cultured for 12 hours with the indicated DC populations.

(a) Scheme of *in vitro* co-culture.

(b) Quantification of CD69 expression.

Statistics were performed with unpaired Student's t test. Error bars represent SEM.

P-values > 0.05: non-significant (ns); P-values ≤ 0.05: significant (*); P-values ≤ 0.01: significant (**); P-values ≤ 0.001: significant (***).

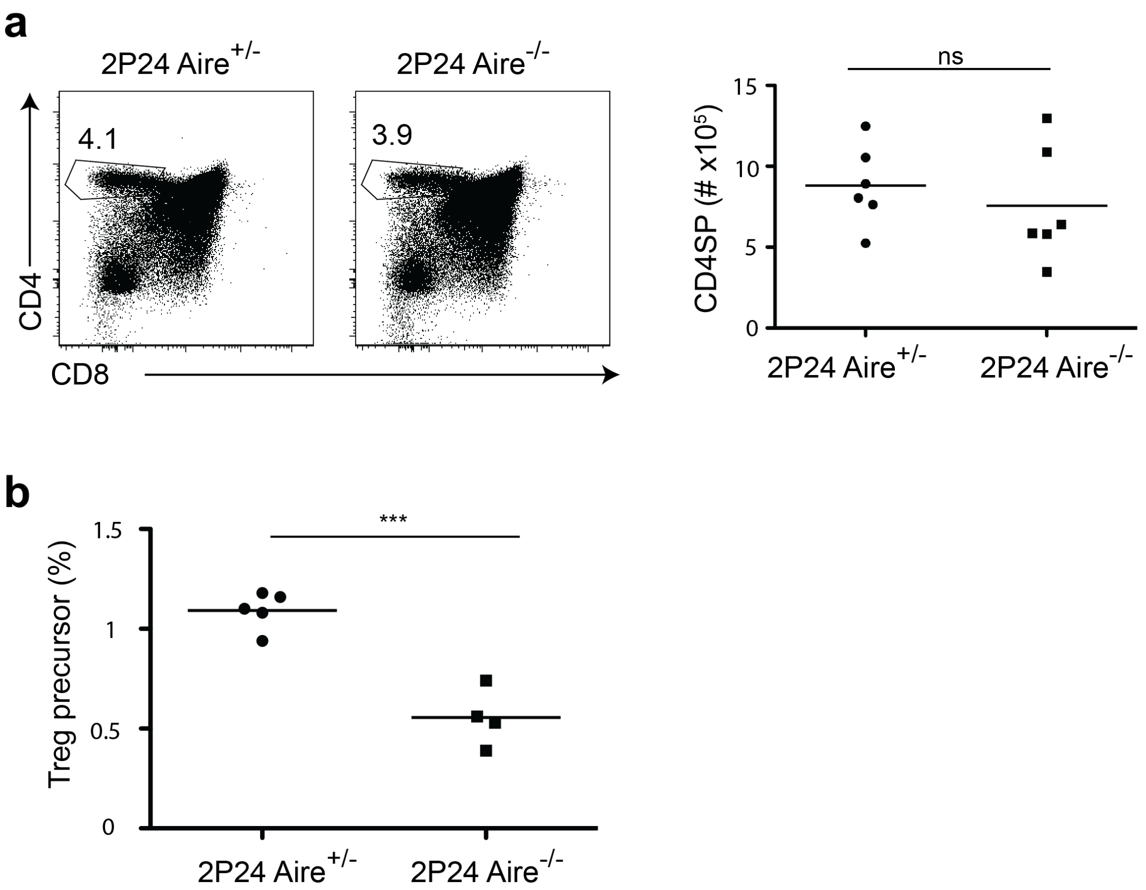

Supplementary Figure 4

Supplementary Figure 4. 2P24 Aire$^{-/-}$ mice display unaffected negative selection and decreased frequency of Treg precursors.

(a) Left two panels: flow cytometry of thymocytes from 2 month-old 2P24 Aire$^{+/-}$ and 2P24 Aire$^{-/-}$ mice, gated on total live cells. Right panel: CD4SP cell numbers from 2 month-old 2P24 Aire$^{+/-}$ and 2P24 Aire$^{-/-}$ mice.

(b) Treg precursor (CD4SP CD25^{+}FOXP3^{-}) frequency in CD4SP FOXP3^{-} cells from 2 month-old 2P24 Aire$^{+/-}$ and 2P24 Aire$^{-/-}$ mice.

Results are the summary of two independent experiments. Statistics were performed with unpaired Student's t test. Error bars represent SEM. P-values > 0.05:

non-significant (ns); P-values ≤ 0.05: significant (*); P-values ≤ 0.01: significant (**); P-values ≤ 0.001: significant (***).

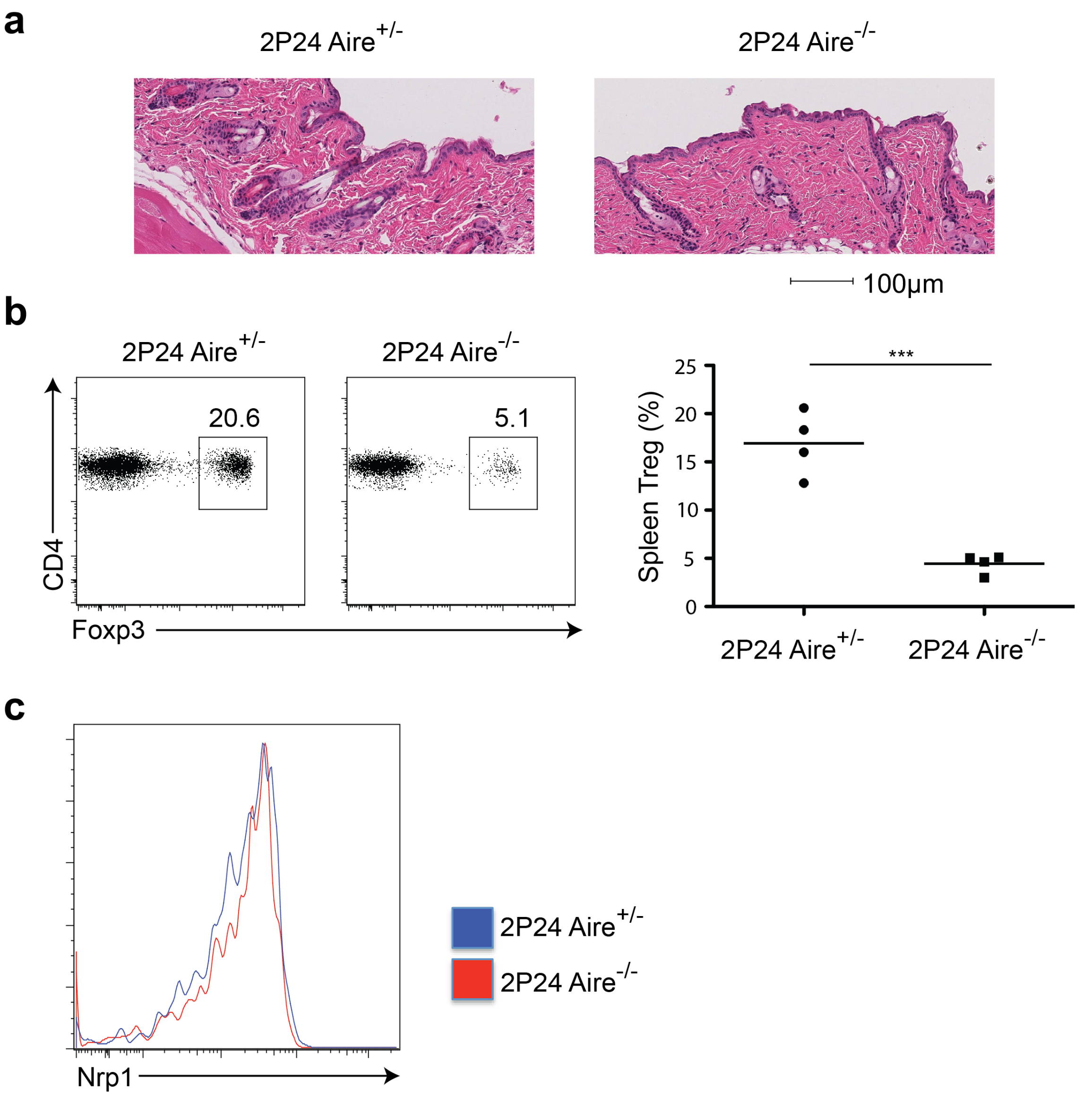

Supplementary Figure 5

Supplementary Figure 5. 2P24 Aire$^{-/-}$ mice harbor a reduced peripheral Treg population compared to 2P24 Aire$^{+/-}$ mice.

(a) Skin H&E staining of 2 month-old 2P24 Aire$^{+/-}$ and 2P24 Aire$^{-/-}$ mice.

(b) Flow cytometry of spleen cells from 2 month-old 2P24 Aire$^{+/-}$ and 2P24 Aire$^{-/-}$ mice; gated on CD4^{+}CD3^{+} cells.

(c) Nrp1 expression on spleen Treg cells from 2P24 Aire$^{+/-}$ and 2P24 Aire$^{-/-}$ mice.

Statistics were performed with unpaired Student's t test. Error bars represent SEM. P-values > 0.05: non-significant (ns); P-values ≤ 0.05: significant (*); P-values ≤ 0.01: significant (**); P-values ≤ 0.001: significant (***).

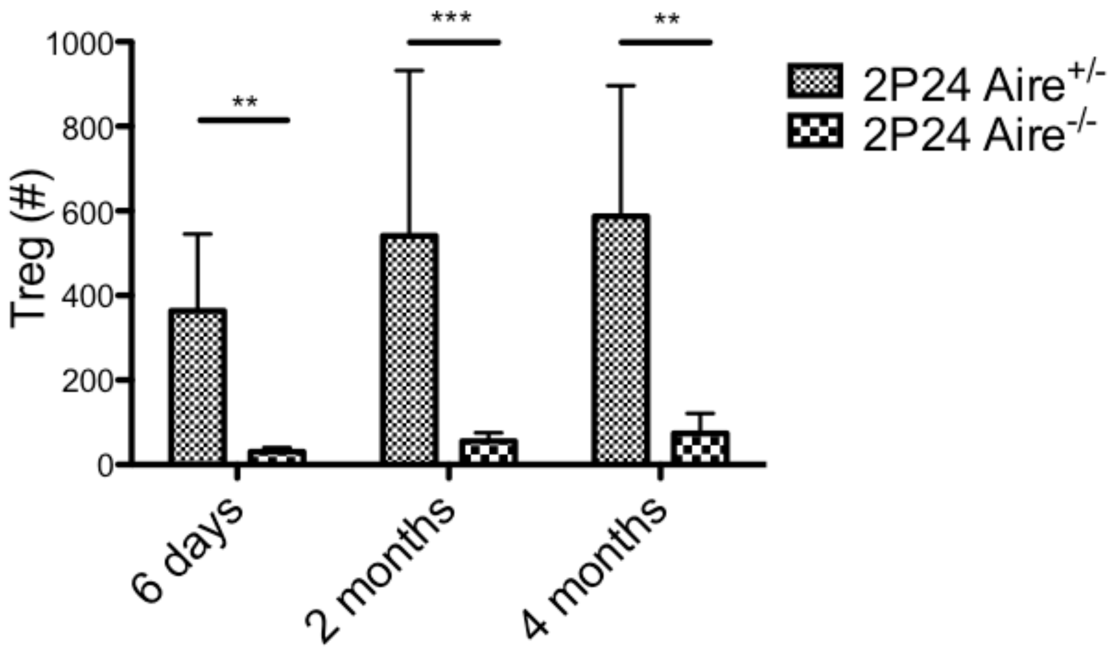

Supplementary Figure 6

Supplementary Figure 6. 2P24 Aire$^{-/-}$ mice harbor a reduced thymic Treg population since the perinatal period, and remain reduced throughout life.

Treg cell number in the thymi of 2P24 Aire$^{+/-}$ and 2P24 Aire$^{-/-}$ mice of different ages. Statistics were performed with unpaired Student's t test. Error bars represent SEM. P-values > 0.05: non-significant (ns); P-values ≤ 0.05: significant (*); P-values ≤ 0.01: significant (**); P-values ≤ 0.001: significant (***).

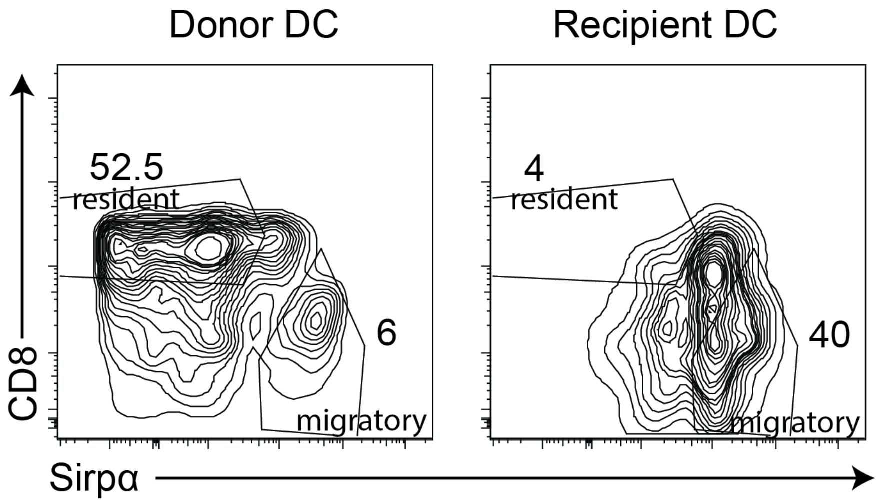

Supplementary Figure 7

Supplementary Figure 7. Migratory and resident DC in transplanted thymi.

Flow cytometry of DC from kidney capsule transplants described in Figure 3. Gated on CD11c⁺ cells.

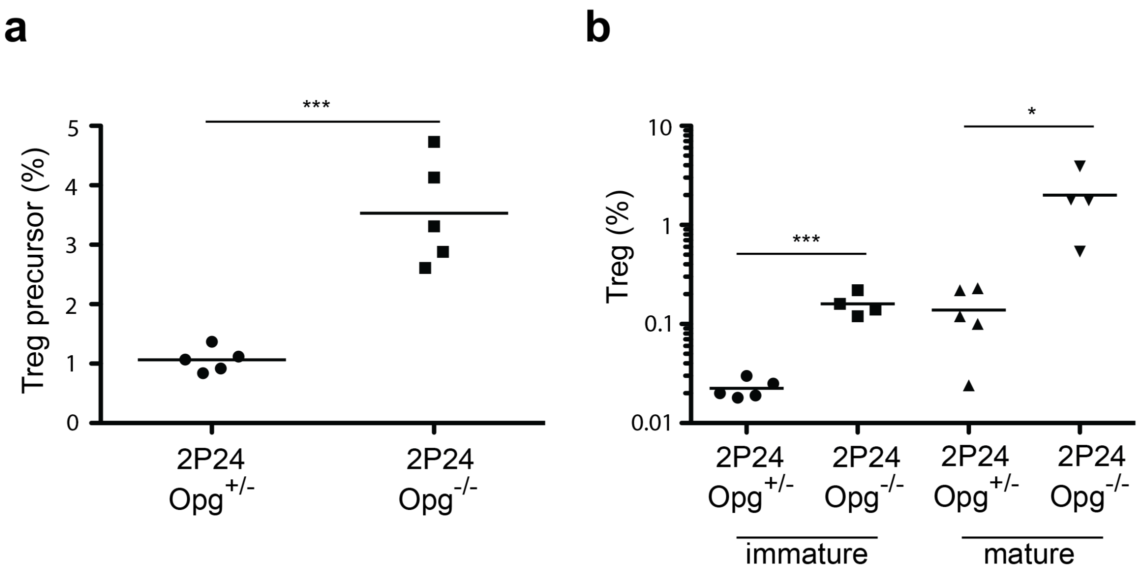

Supplementary Figure 8

Supplementary Figure 8. 2P24 Opg$^{-/-}$ mice have increased Treg cells at all developmental stages.

(a) Treg precursor (CD4SP CD25^{+}FOXP3^{-}) frequency in CD4SP FOXP3^{-} cells from 2 month-old 2P24 Opg$^{+/-}$ and 2P24 Opg$^{-/-}$ mice.

(b) Treg (CD4SP CD25^{+}FOXP3^{+}) frequency in immature (CD62L^{-}CD69^{+}) and mature (CD62L^{+}CD69^{-}) CD4SP cells from 2 month-old 2P24 Opg$^{+/-}$ and 2P24 Opg$^{-/-}$ mice.

Results are the summary of two independent experiments.

Statistics were performed with unpaired Student's t test. Error bars represent SEM.

P-values > 0.05: non-significant (ns); P-values ≤ 0.05: significant (*); P-values ≤ 0.01: significant (**); P-values ≤ 0.001: significant (***).

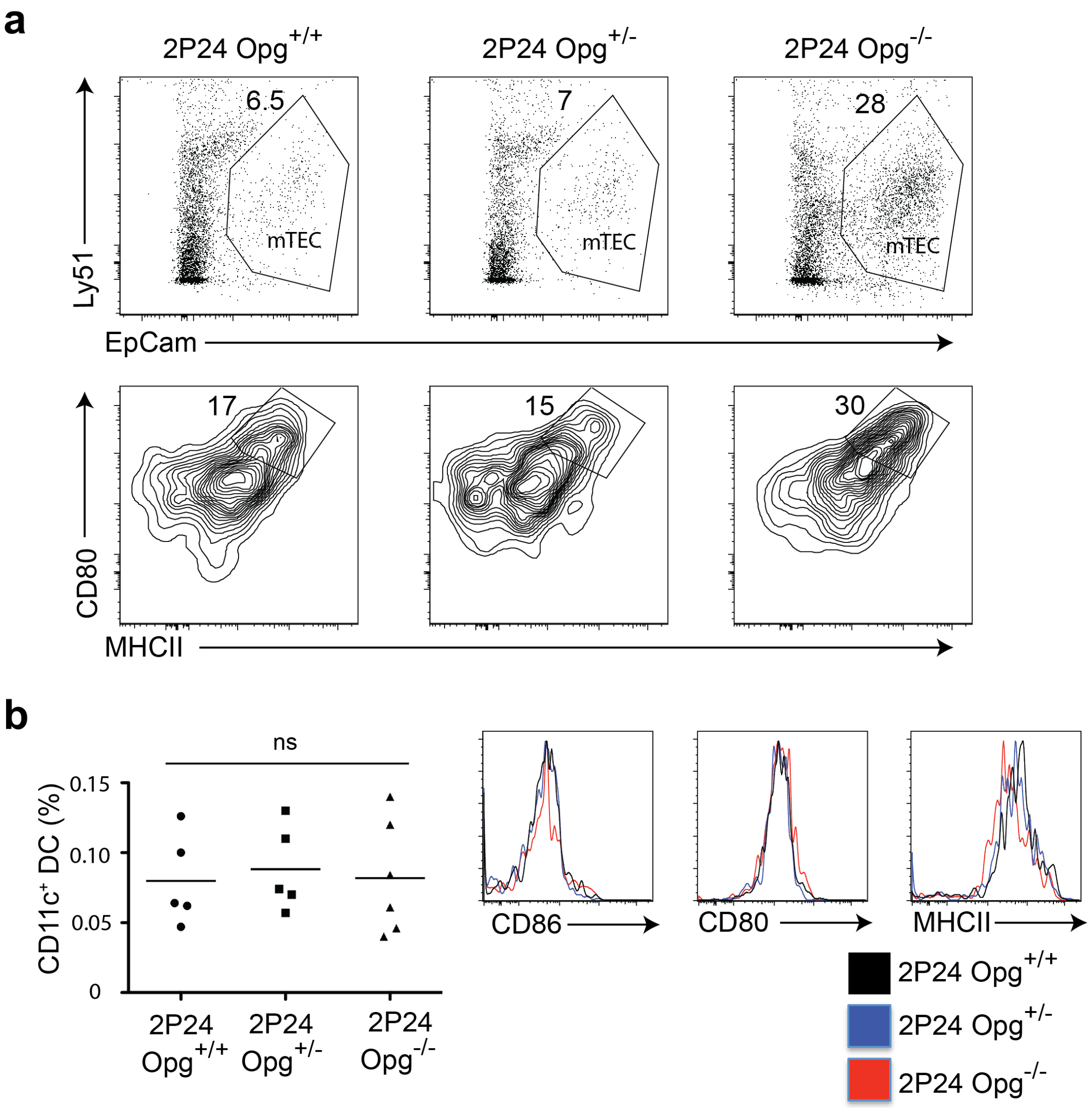

Supplementary Figure 9

Supplementary Figure 9. Epithelial cell and DC characterization in 2P24 Opg$^{-/-}$ mice.

Thymi of 2P24 Opg$^{+/+}$, 2P24 Opg$^{+/-}$ and 2P24 Opg$^{-/-}$ mice were digested with dispase and collagenase to release epithelial cells and DC, which were analyzed by flow cytometry.

(a) Epithelial cell analysis. Top three panels: Analysis of $CD45^-$ cells. Shown inside the $EpCam^+Ly51^-$ gate are the medullary epithelial cells. Bottom three panels: Analysis of maturation state of medullary epithelial cells gated as in top three panels. Mature mTECs are shown as $MHCII^{hi}$ $CD80^{hi}$.

(b) DC analysis. Left panel: DC percentage in thymi of 2P24 $Opg^{+/+}$, 2P24 $Opg^{+/-}$ and $Opg^{-/-}$ mice.

Right three panels: Activation state of DC as shown by CD80, CD86 and MHCII expression.

Data are representative of two independent experiments with three to four mice per group. Statistics were performed with unpaired Student's t test. Error bars represent SEM. P-values > 0.05: non-significant (ns); P-values ≤ 0.05: significant (*); P-values ≤ 0.01: significant (**); P-values ≤ 0.001: significant (***).

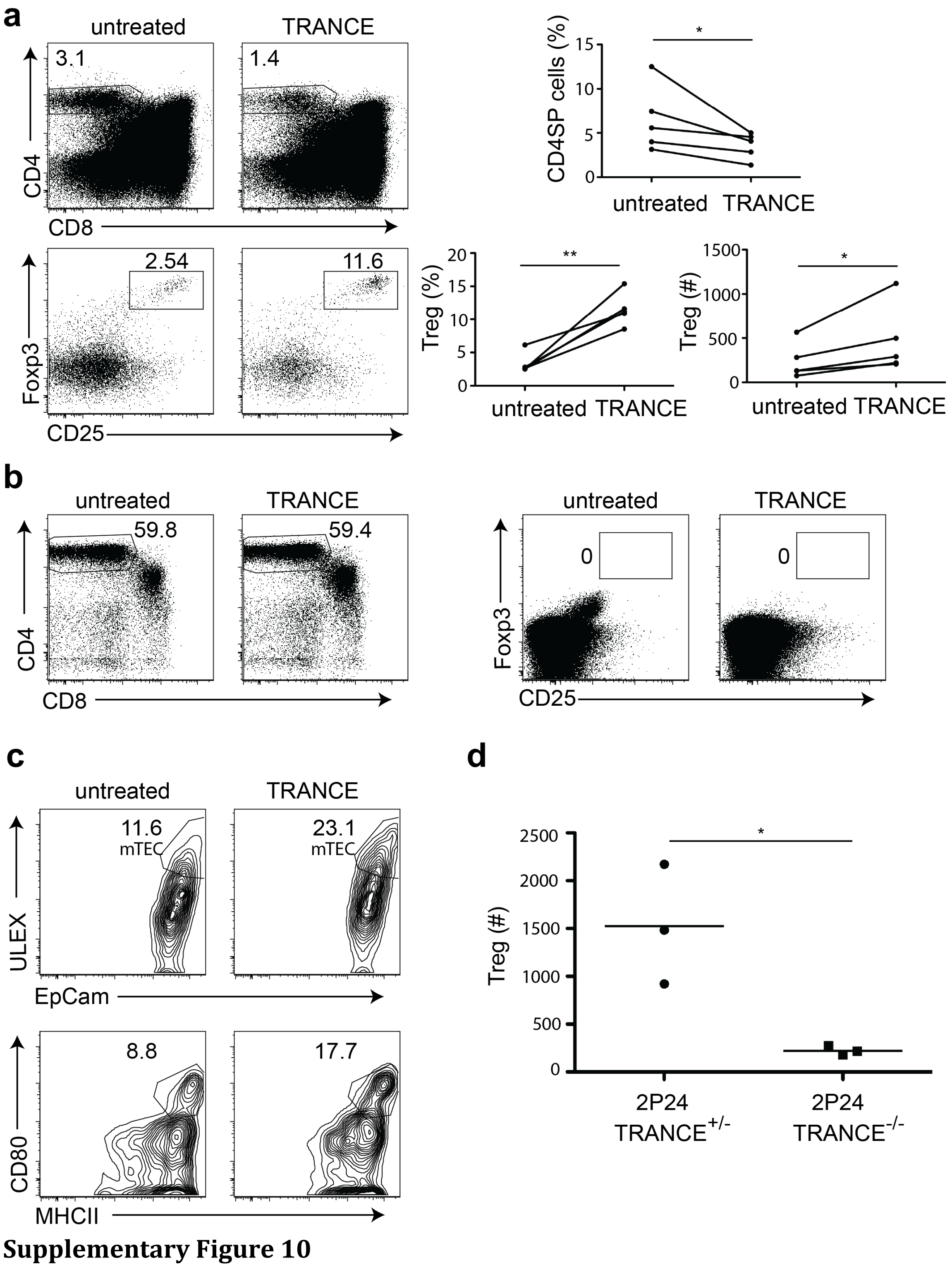

Supplementary Figure 10

Supplementary Figure 10. The role of TRANCE in 2P24 Treg generation.

(a) and (b): TRANCE administration increases Treg generation in 2P24 (Treg TCR) FTOC but not anti-MBP (Tconv TCR) FTOC.

FTOC were set up as described in Fig3. One lobe of each pair was treated with 300ng/ml TRANCE while the other one was cultured with medium only. Seven days later, the cultured lobes were analyzed by flow cytometry.

(a) 2P24 FTOC. Left panels: representative flow cytometry plots. Top two panels: gated on total live cells; Bottom two panels: gated on CD4SP cells.

Right Panels: CD4SP cell frequency, Treg frequency and Treg cell numbers of the paired FTOC. Results are representative of three independent experiments.

(b) anti-MBP TCR FTOC. Left two panels: gated on total live cells; Right two panels: gated on CD4SP cells. Results are representative of two independent experiments.

(c) Epithelial cell staining of FTOC. Upper two panels: gated on $CD45^{-}EpCam^{+}$ cells. Medullary epithelial cells are identified as $EpCam^{+}ULEX^{+}$ cells. Lower panels: maturation state of mTECs, gated on $EpCam^{+}ULEX^{+}$ mTECs. Results are representative of three independent experiments.

(d) Reduction of 2P24 Treg cells in TRANCE KO mice. 2P24 mice were crossed with $TRANCE^{-/-}$ mice and thymi from 2 week-old 2P24 $TRANCE^{+/-}$ and $TRANCE^{-/-}$ mice were analyzed by flow cytometry. Shown is Treg cell number per total thymi. Results are the summary of two independent experiments.

Statistics in (a) were performed with paired Student's t test and statistics in (d) were performed with unpaired Student's t test. Error bars represent SEM. P-values > 0.05: non-significant (ns); P-values ≤ 0.05: significant (*); P-values ≤ 0.01: significant (**); P-values ≤ 0.001: significant (***).

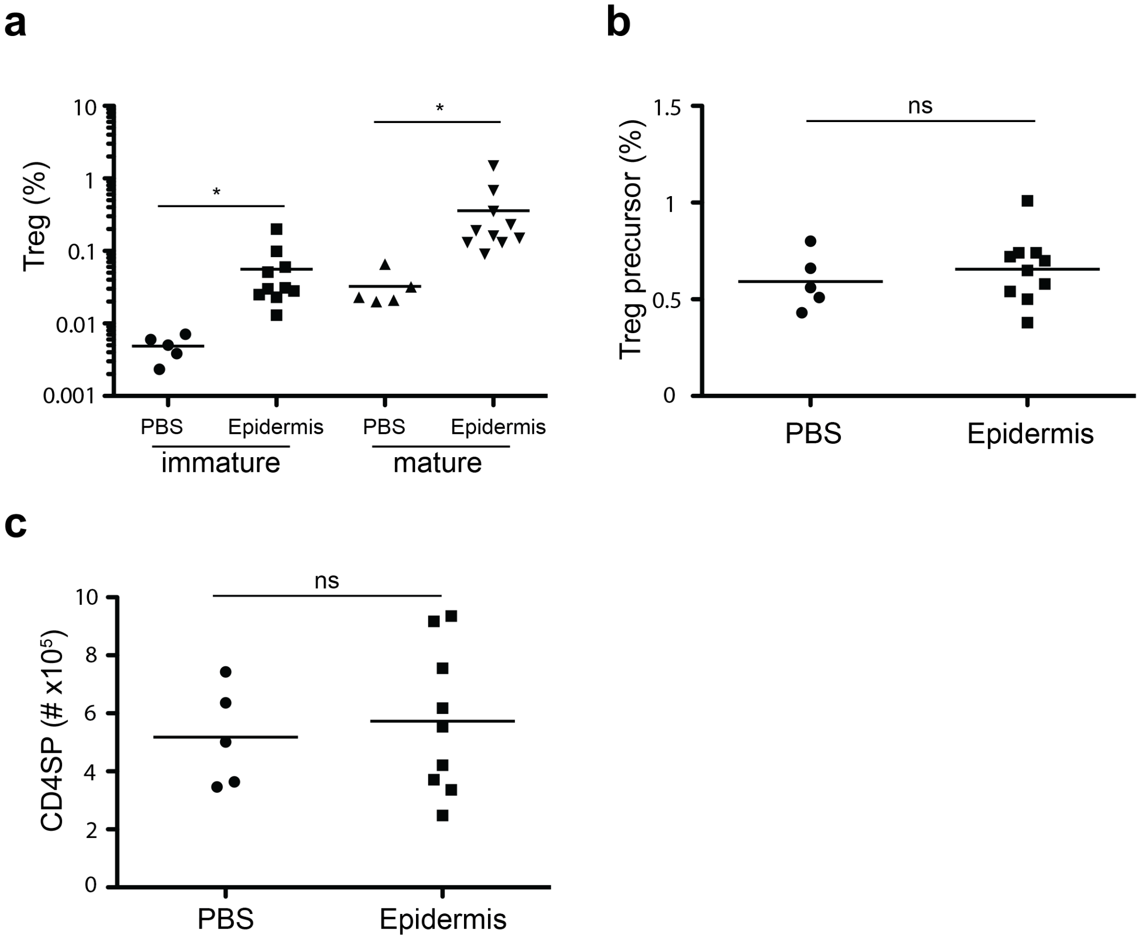

Supplementary Figure 11.

Supplementary Figure 11. Intrathymic injection of epidermis lysate increases mature and immature Treg cells without affecting the negative selection of CD4SP cells in 2P24 Aire$^{-/-}$ mice.

(a) Treg (CD4SP CD25$^+$FOXP3$^+$) frequency in immature (CD62L$^-$CD69$^+$) and mature (CD62L$^+$CD69$^-$) CD4SP cells from 2P24 Aire$^{-/-}$ mice injected with epidermis lysate.

(b) Treg precursor (CD4SP CD25$^+$FOXP3$^-$) frequency in CD4SP FOXP3$^-$ cells from 2P24 Aire$^{-/-}$ mice injected with epidermis lysate.

(c) CD4SP cell numbers from 2P24 $Aire^{-/-}$ mice injected with epidermis lysate.

Statistics were performed with unpaired Student's t test. Error bars represent SEM.

P-values > 0.05: non-significant (ns); P-values ≤ 0.05: significant (*); P-values ≤ 0.01: significant (**); P-values ≤ 0.001: significant (***).
